# A Combination of Let-7d, Let-7g and Let-7i Serves as a Stable Reference for Normalization of Serum microRNAs

**DOI:** 10.1371/journal.pone.0079652

**Published:** 2013-11-05

**Authors:** Xi Chen, Hongwei Liang, Danping Guan, Cheng Wang, Xiaoyun Hu, Lin Cui, Sidi Chen, Chunni Zhang, Junfeng Zhang, Ke Zen, Chen-Yu Zhang

**Affiliations:** 1 Jiangsu Engineering Research Center for microRNA Biology and Biotechnology, State Key Laboratory of Pharmaceutical Biotechnology, School of Life Sciences, Nanjing University, Nanjing, Jiangsu, China; 2 Nanjing MicroMedMark Biotech Co., Ltd., Nanjing, Jiangsu, China; 3 Beijing MicroMedMark Biotech Co., Ltd., Beijing, China; 4 Department of Clinical Laboratory, Jinling Hospital, Clinical School of Medical College, Nanjing University, Nanjing, Jiangsu, China; The University of Kansas Medical center, United States of America

## Abstract

Recent studies have indicated that circulating microRNAs (miRNAs) in serum and plasma are stable and can serve as biomarkers of many human diseases. Measurement of circulating miRNAs with sufficient sensitivity and precision, however, faces some special challenges, among which proper normalization is the most critical but often an underappreciated issue. The primary aim of this study was to identify endogenous reference genes that maintain consistent levels under various conditions to serve as an internal control for quantification of serum miRNAs. We developed a strategy combining Illumina’s sequencing by synthesis (SBS) technology, reverse transcription quantitative polymerase chain reaction (RT-qPCR) assay, literature screening and statistical analysis to screen and validate the most suitable reference genes. A combination of let-7d, let-7g and let-7i is selected as a reference for the normalization of serum miRNAs and it is statistically superior to the commonly used reference genes U6, RNU44, RNU48 and miR-16. This has important implications for proper experimental design and accurate data interpretation.

## Introduction

MicroRNAs (miRNAs) are small noncoding RNAs with a length of approximately 22 nucleotides that play important roles in gene regulatory networks [1-3]. Recently, we and other groups have demonstrated that miRNAs circulate in a highly stable, cell-free form in body fluids including serum [4,5], plasma [6], saliva [7], urine [8] and milk [9,10]. Furthermore, aberrant expression of circulating miRNAs has been detected in a wide range of pathological conditions including cancer [4-8], diabetes [5], acute myocardial infarction [11] and tissue injury [12]. These findings suggest broad opportunities for development of circulating miRNAs as non-invasive biomarkers for molecular diagnostics and prognostics. However, when performing the experiments to quantify circulating miRNAs, variations in the amount of starting material, sample collection, RNA extraction and enzymatic efficiency may introduce potential bias and contribute to quantification errors. Given these concerns, the development of an effective normalization strategy is critical for evaluating circulating miRNAs. Among a variety of available normalization methods, normalization against a stable reference gene (or better a set of multiple stable reference genes) is currently the most accurate and suitable method for evaluation of circulating miRNAs [13]. In contrast, other normalization methods may obscure real changes and produce artificial changes. For example, data normalization can be carried out against total RNA content, but this approach requires an accurate quantification of the isolated total RNA. Because serum and plasma are cell-free samples with very low amounts of total RNA (the concentration of total RNA purified from serum/plasma can be measured using NanoDrop spectrophotometer but is usually < 50 ng/μL) [14], standard methods for measurement of the RNA yield and quality are inappropriate for these types of samples. This means that normalization to total RNA is not a reliable and accurate method. 

Here, we report a systematic strategy to identify and characterize the most stable reference genes for normalizing serum miRNAs in healthy people and patients with a variety of diseases. A final combination of three miRNAs, let-7d, let-7g and let-7i, was found to give highly consistent results across numerous healthy controls and patients with diseases. These miRNAs were statistically superior to the most commonly used reference genes in the quantification of serum miRNAs.

## Methods

### Patients and Control Subjects

Patients with pathologically confirmed, newly diagnosed, untreated cancers, inflammatory diseases or type 2 diabetes were recruited at the ***Jinling*** Hospital (Nanjing, China). Blood was also collected from healthy participants during physical examinations performed at the Jinling Hospital. Written informed consent was obtained from all patients and healthy participants prior to the study. The study protocol was approved by the ethics committee of Nanjing University. Sample sets were shown in Table 1.

**Table 1:** Demographic and clinical features of patients and healthy controls.

	**Sample set 1**			**Sample set 2**			**Sample set 3**			**Sample set 4**		
**Variable**	Patients (n=130)	Controls (n=100)	*p*-value	Patients (n=21)	Controls (n=35)	*p*-value	Patients (n=777)	Controls (n=1278)	*p*-value	Patients (n=84)	Controls (n=41)	*p*-value
**Average age (years)**	60.77±9.42	36.51±22.65	< 0.001^a^	63.24±7.32	60.69±8.44	0.2553^a^	59.29±9.96	59.19±10.10	0.8212^a^	60.88±8.60	58.63±9.51	0.1878^a^
**Age (years)**			0.0035^b^			0.1470^b^			0.6890^b^			0.2764^b^
≤ 60	65	70		7	20		394	661		41	25	
> 60	65	30		14	15		383	617		43	16	
**Sex**			0.9079^b^			0.9432^b^			0.9023^b^			0.5604^b^
Male	66	50		13	22		464	768		43	24	
Female	64	50		8	13		313	510		41	17	
**Histological types**												
NSCLC	30			11			28			18		
Pancreatic cancer	10			10			56			12		
Gastric cancer	20						47			20		
Esophageal cancer	20						16			10		
Colorectal cancer	10						20					
HCC	10						20					
Breast cancer	20						20			18		
Ovarian cancer							20			6		
Cervical cancer	10						30					
Nephritis							101					
Colitis							73					
Pancreatitis							18					
Pneumonia							8					
Type 2 diabetes							320					

^a^ t-test; ^b^ two-sided λ^2^ test. NSCLC, Non-small cell lung cancer; HCC, Hepatocellular carcinoma.

### RNA Isolation, SBS Technology and RT-qPCR

Venous blood samples (~5 ml) were collected from each donor and placed in a serum separator tube. Samples were processed within one hour. Separation of the serum was accomplished by centrifugation at 800 g for 10 min at room temperature, followed by a 15-min high-speed centrifugation at 10,000 g at room temperature to completely remove the cell debris. The supernatant serum was recovered and stored at -80°C until analysis. Those serum samples with pink/red discolouration were considered to be haemolysed and were excluded.

For the Illumina’s sequencing by synthesis (SBS) technology, serum pools were created by combining 10 samples (5 mL each) and mixing vigorously. Then, total RNA was extracted from the 50 mL of pooled serum using the TRIzol Reagent (Invitrogen, Carlsbad, CA, USA) according to the manufacturer’s instructions. Then sequencing procedure was conducted as previously described [5]. For detailed methodology, see Method S1.

For the reverse transcription quantitative polymerase chain reaction (RT-qPCR) assay, total RNA was extracted from 100 μL of serum with a one-step phenol/chloroform purification protocol. Quantification of serum miRNAs was then carried out using a Taqman miRNA PCR kit (Applied Biosystems, Foster City, CA, USA) according to the manufacturer’s instructions. Primers used in this study are shown in Table S1. For detailed methodology, see Method S1.

### Data analysis

The RT-qPCR was performed in triplicate, and each experiment was repeated several times. Data shown are presented as the mean ± SEM of at least three independent experiments. Statistical analyses were performed with SPSS 15.0 statistical software, and a p-value < 0.05 using t-test was considered statistically significant. Selection of optimal reference gene was conducted using geNorm and NormFinder as previously described [15,16]. For detailed methodology, see Method S1.

## Results

### Study Design

**Figure 1:**
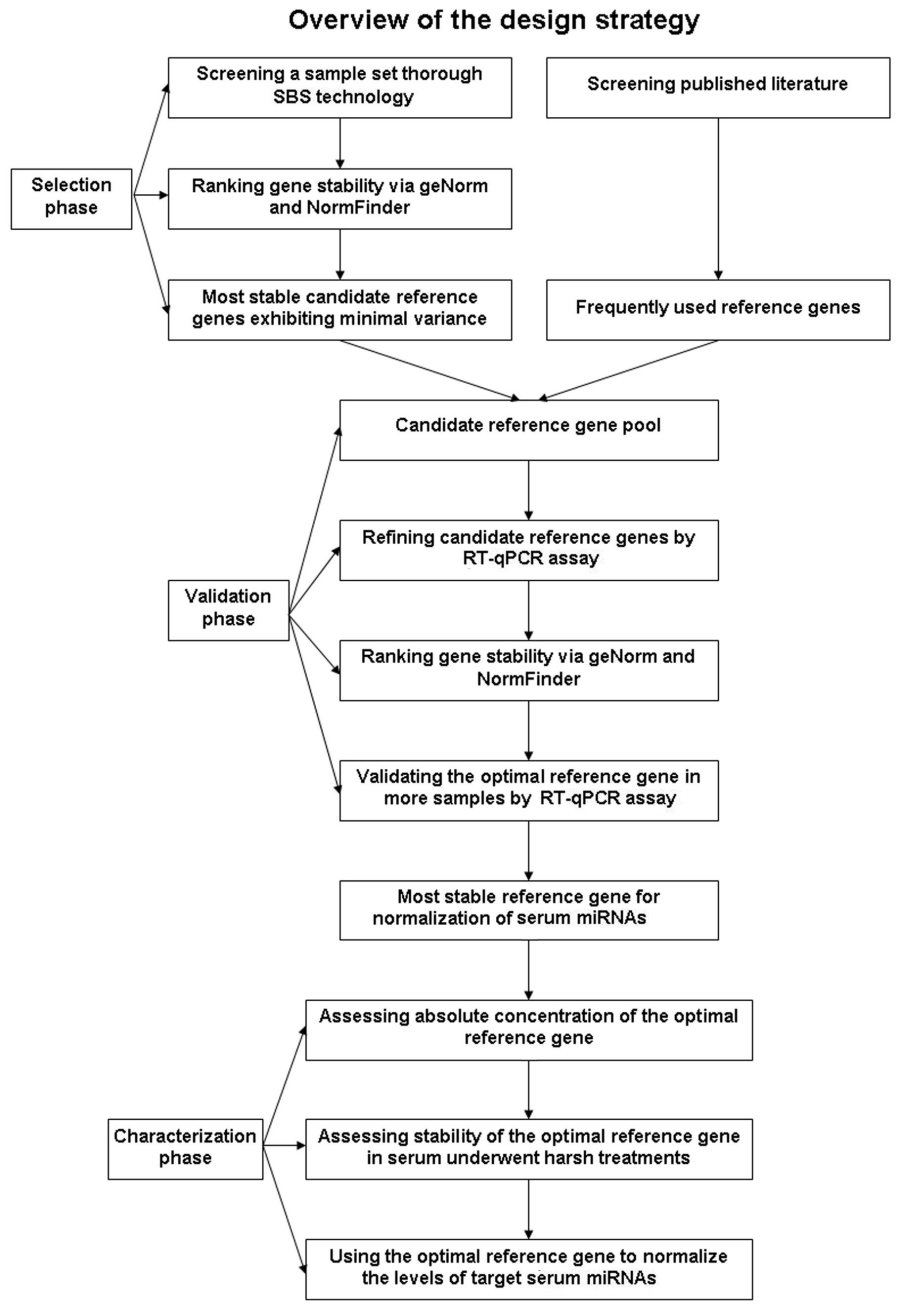
Strategy for the identification of stable reference genes for normalizing serum miRNAs.

We developed a strategy combining four main steps to identify and validate the suitable set of genes for normalization of serum miRNAs (Figure 1). In step one, we employed SBS technology to screen a sample set representing a wide range of physiological and pathological conditions. Two statistical algorithms, geNorm and NormFinder, were implemented to rank gene stability and select the most stable candidates exhibiting minimal variance across different samples. The second step involved screening published literature to identify frequently used reference genes. This two-step approach to selecting candidate reference genes provided more gene information in order to find optimal reference genes with greater accuracy. In step three, the selected candidates identified in steps one and two were combined, and their stability was evaluated by RT-qPCR and analyzed by geNorm and NormFinder in serum samples from 21 cancer patients and 35 controls. The most stable reference gene identified was subsequently validated in serum samples from an additional 1278 controls, 257 cancer patients, 200 patients with inflammatory diseases and 320 patients with type 2 diabetes. Finally, we characterized the absolute concentration of the optimal reference genes identified in our primary analysis and assessed their stability in serum after various treatments. In an independent experiment, we used the optimal reference genes to normalize the levels of target miRNAs in serum samples from 84 cancer patients and 41 controls, demonstrating that the reference gene selection can have a significant influence on serum miRNA quantification.

### Selection of the Most Stable Reference Genes by SBS Technology

**Figure 2:**
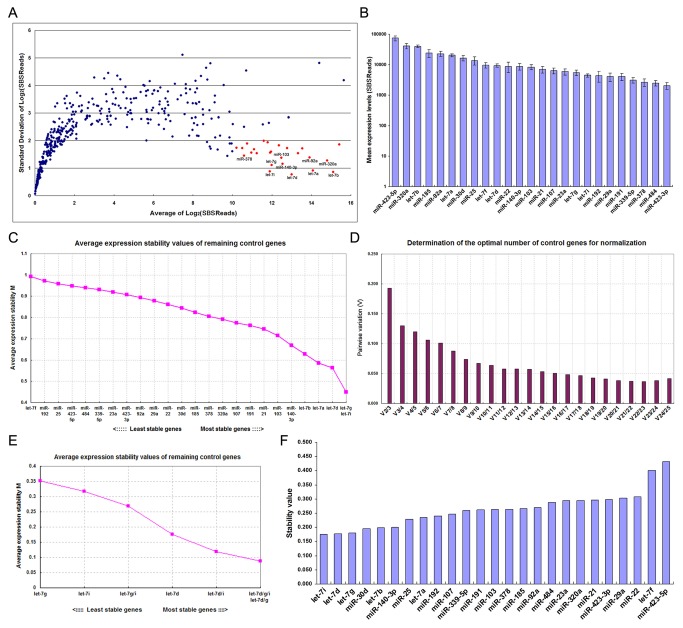
Selection of the most stable reference genes by SBS technology. (**A**) Sera from cancer patients and healthy participants were pooled separately as described above, and miRNA levels were determined using SBS technology. SBS reads were converted to the log_2_ scale. The average log_2_-transformed read of each miRNA was plotted against the standard deviation of the log_2_-transformed read. MiRNAs highlighted in red are those with higher abundance (log_2_-transformed reads > 10) and lower standard deviations (< 1) in the dataset. (**B**) The average expression values (SBS reads ± standard errors) of the selected miRNAs were plotted. (**C**) Selection of the most stable reference genes from a panel of 25 genes using geNorm. The geNorm program calculates the average expression stability value (M) for each gene. Genes with the lowest M values are considered the most stable. The least stable gene with the highest M value was automatically excluded for the next calculation round. The x-axis from left to right indicates the ranking of the reference genes according to their expression stability from the least to the most stable, and the y-axis represents the M values of the remaining reference genes. (**D**) Identification of the optimal number of reference genes for accurate normalization using geNorm. V is the pairwise variation (V_n_/V_n+1_) between two sequential normalization factors (NF_n_ and NF_n+1_). The magnitude of the change in the normalization factor after the inclusion of an additional reference gene reflects the improvement that is obtained. The authors of geNorm suggest that V > 0.15 should be considered the threshold for including an extra reference gene in the assay, and the least number of genes for each V < 0.15 is selected as the optimal set of genes for normalization. (**E**) Selection of the most stable reference gene or gene combinations using geNorm. In this case, geNorm indicated that the combination of let-7d, let-7g and let-7i was statistically superior to other combinations or each individually. (**F**) Identification of the most stable reference genes using NormFinder. The NormFinder algorithm ranks the set of candidate normalization genes according to their expression stability in different groups (e.g., disease versus normal). According to this algorithm, lower stability values of the individual genes indicate greater gene stability. In this case, 23 samples were divided into two groups (12 normal controls and 11 cancer patients). Blue bars represent the stability values of the candidate genes.

We first screened a SBS dataset to identify stable serum miRNAs across various physiological and pathological conditions. A total of 23 pooled serum samples were analyzed, including 8 healthy male or female samples of different ages (baby boy, baby girl, young boy, young girl, middle-aged man, middle-aged woman, old man and old woman; each pool was created by combining 10 individual serum samples), 2 mixed healthy samples (middle-aged and old; each pooled from 5 male and 5 female) and 13 cancer patients (3 non-small cell lung cancer, 2 breast cancer, 2 gastric cancer, 2 esophageal cancer, 1 colorectal cancer, 1 pancreatic cancer, 1 cervical cancer and 1 hepatocellular carcinoma; each pool was created by combining 10 individual serum samples). MiRNAs were considered stable if they fulfilled the following criteria: (1) expressed in all samples; (2) highly expressed, as measured by the mean; and (3) consistently expressed, as measured by the standard deviations. According to these criteria, 25 miRNAs were selected as candidate reference genes. As shown in Figure 2A, SBS reads were converted to the log_2_ scale, and genes were sorted by the mean expression levels and standard deviations. Among the miRNAs detected, 25 miRNAs had high abundance (log_2_-transformed reads > 10) and low standard deviations (

< 1) in the dataset. The mean expression values of the selected 25 miRNAs are shown in Figure 2B. 

The stability of the candidate reference genes was further evaluated by two different algorithms, geNorm [15] and NormFinder [16]. The geNorm algorithm calculates the average expression stability (M value) of a gene by using pairwise comparisons, ranking putative reference genes according to the similarity of expression profiles across a sample set [15]. Genes with the lowest M values are considered the most stable. The stability ranking of each candidate gene is determined by stepwise exclusion of the gene with highest M value, followed by recalculation of average expression stability for the remaining genes until the two most stable genes are found [15]. The curve presented in Figure 2C plots the average expression stability of the 25 candidate reference genes. As shown, let-7g was the most stably expressed gene within the group, followed by let-7i and let-7d. The geNorm analysis also allows for evaluation of the suitable number of reference genes required for reliable and accurate normalization [15]. This algorithm determines the optimal number of reference genes using a metric called V, which is the pairwise variation (V_n_/V_n+1_) of two sequential normalization factors (NF_n_/NF_n+1_). It is suggested that the cut-off value of 0.15 should be considered as a limit beneath which the use of additional reference genes would not be required [15]. The results showed that the optimal number of genes for accurate normalization was three, and the combination of let-7d, let-7g and let-7i was sufficient to accurately normalize a target gene in this dataset, yielding a V value of 0.13 that is lower than the cutoff value 0.15 (Figure 2D). Indeed, the total amount of let-7d/g/i trio was optimal for normalization and was statistically superior to the combinations of two let-7 members or individual let-7 member (Figure 2E). On the other hand, NormFinder uses a solid statistical framework to estimate not only the overall expression variation of the candidate reference genes, but also the variation between subgroups (e.g., tumor versus normal) [16]. According to this algorithm, genes with the lowest stability will be ranked highest [16]. When the gene expression stability was estimated independently using the NormFinder software, the result was essentially the same as that from geNorm. The NormFinder algorithm selected let-7i as the optimal reference gene for normalization, followed by let-7d and let-7g (Figure 2F).

### Literature-based selection of additional candidate reference genes

The three newly selected candidate reference genes were further evaluated along with several traditionally utilized reference genes, including large molecular weight RNAs (GAPDH and β-actin), small nuclear/nucleolar RNAs (snRNA/snoRNA) (U6, RNU44 and RNU48) and housekeeping miRNAs (miR-16, miR-191, miR-103 and miR-23a). GAPDH and β-actin were selected based on previous studies reporting their stable expression in tissues and cells [17]. U6 [18-20], RNU44 [21-23], RNU48 [24,25], miR-16 [26], miR-191 [27], miR-103 [27] and miR-23a [28] were selected because they are commonly utilized reference genes in analyzing tissue/cell miRNA. In addition, we focused on U6 [29,30] and miR-16 [4,31-33], which have been used as reference genes for normalization of serum miRNAs.

### Development of an approach to measure the total amount of let-7d, let-7g and let-7i in a run

The RT-qPCR assay is currently the most sensitive and reliable method to determine the levels of circulating miRNAs [13,34]. However, while SBS technology is easy to assess the total amount of let-7d, let-7g and let-7i in a run, in the RT-qPCR assay one reaction just enable analysis of one miRNA. Therefore, to provide increased flexibility and decreased time and effort for determining the reference gene, an approach should be developed to simultaneously analyze multiple members of the let-7d/g/i trio. Given that the TaqMan miRNA assays can successfully discriminate each members of the let-7 family without interference (Figure S1) and that each member of the let-7d/g/i trio has approximately equal amplification efficiencies (Figure S2), we designed an approach to simultaneously measure the total amount of let-7d/g/i trio in a same RT-qPCR reaction. Briefly, let-7d, let-7g and let-7i in 5 μL of total RNA were reverse-transcribed in a single reaction using specific RT Primer pool, a mixture of stem-loop primers of let-7d, let-7g and let-7i (in the ratio of 1:1:1). Accordingly, real-time PCR was performed using TaqMan miRNA probe pool of let-7d, let-7g and let-7i (in the ratio of 1:1:1). With this approach, one reaction was sufficient to accurately quantify the total amount of let-7d/g/i trio, no matter how many individual let-7d, let-7g and let-7i were present in the samples (Table 2).

**Table 2:** Quantification of the total amount of let-7d, let-7g and let-7i in different let-7d/g/i mixture.

miRNA input (attomole)			Total let-7d/g/i amount	
let-7d	let-7g	let-7i	Cq^1^	SD^2^
10	0	0	23.447	0.202
0	10	0	23.558	0.145
0	0	10	23.527	0.029
6.67	3.33	0	23.358	0.091
6.67	0	3.33	23.484	0.089
3.33	0	6.67	23.259	0.150
3.33	6.67	0	23.340	0.085
0	6.67	3.33	23.229	0.064
0	3.33	6.67	23.349	0.030
3.33	3.33	3.33	23.444	0.024

^1^Cq (quantification cycle), or named Ct (threshold cycle), is defined as the number of cycles required for the fluorescent signal to cross the threshold.

^2^SD, standard deviation

### Validation of the stability of the selected candidate reference genes by RT-qPCR

**Figure 3:**
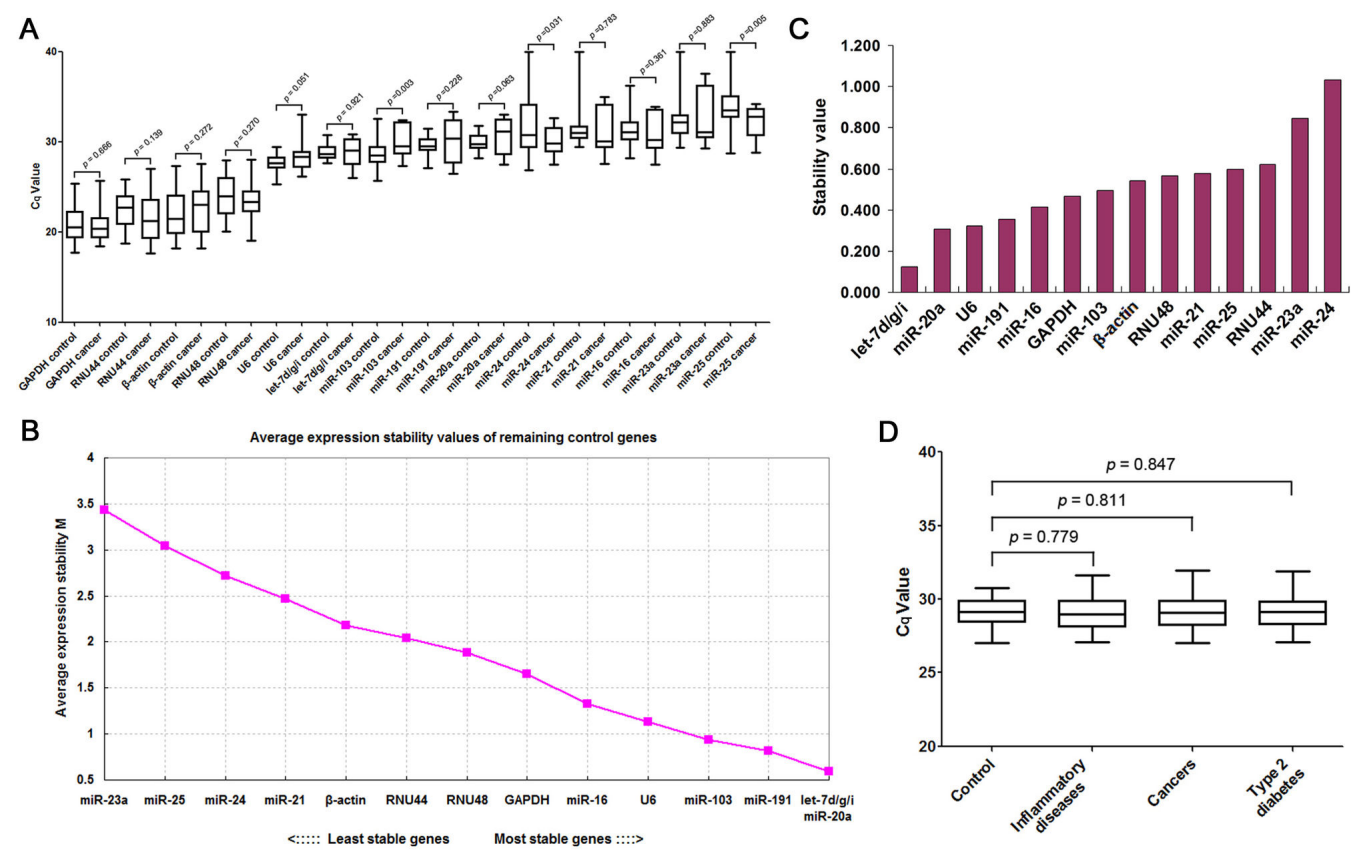
Validation of the stability of the selected reference genes by RT-qPCR assay. (**A**) Expression levels of candidate reference genes in serum. The expression levels of 14 candidates were measured by RT-qPCR in the sera from cancer patients (n = 21) and healthy controls (n = 35). The C_q_ values (median and range) of each miRNA were shown by box-and-whisker plots. Significance was calculated by t-test. (**B**) Identification of the optimal number of reference genes for accurate normalization using geNorm. (**C**) Identification of the most stable reference genes using NormFinder. (**D**) Expression levels of let-7d/g/i in large numbers of serum samples. The expression levels of let-7d/g/i were measured in the sera from healthy controls (n = 1278), cancer patients (n = 254), patients with inflammatory diseases (n = 200) and patients with type 2 diabetes (n = 320). The C_q_ values (median and range) in each group were shown by box-and-whisker plots. Significance was calculated by t-test.

Next, RT-qPCR assay was performed to further evaluate the expression patterns of the selected candidate reference genes in a sample set of 21 cancer patients and 35 healthy controls. As controls, we analyzed miR-20a [35], miR-21 [33,36,37], miR-24 [35] and miR-25 [35,37], the levels of which have previously been shown to be significantly dysregulated in serum from cancer patients. As shown in Figure 3A, the reference genes displayed a wide range of levels, with threshold cycle (Cq) values ranging from 21 to 33, and the smallest variation was observed with let-7d/g/i. Next, the geNorm and NormFinder algorithms were used to rank the candidate reference genes according to their expression stability. Based on calculations performed with geNorm, let-7d/g/i had the most stable expression levels and thus was selected as the best combination of reference genes (Figure 3B). In contrast, miR-191, miR-103, U6, miR-16, RNU48 and RNU44, the commonly used reference genes for miRNA RT-qPCR experiments, ranked behind let-7d/g/i (Figure 3B), suggesting that they should not be considered reliable reference genes for data normalization. NormFinder confirmed the results obtained by geNorm, showing that let-7d/g/i was the most stable reference gene set, whereas miR-24 was the least stable gene (Figure 3C). The identified optimal reference gene set, let-7d/g/i, was subsequently validated in a large sample set containing 1278 healthy controls, 257 cancer patients, 200 patients with inflammatory diseases and 320 patients with type 2 diabetes. As shown in Figure 3D, the expression levels of let-7d/g/i remained constant across individuals tested, independent of the disease condition.

### Characterization of the absolute concentration of let-7d/g/i in serum

**Figure 4:**
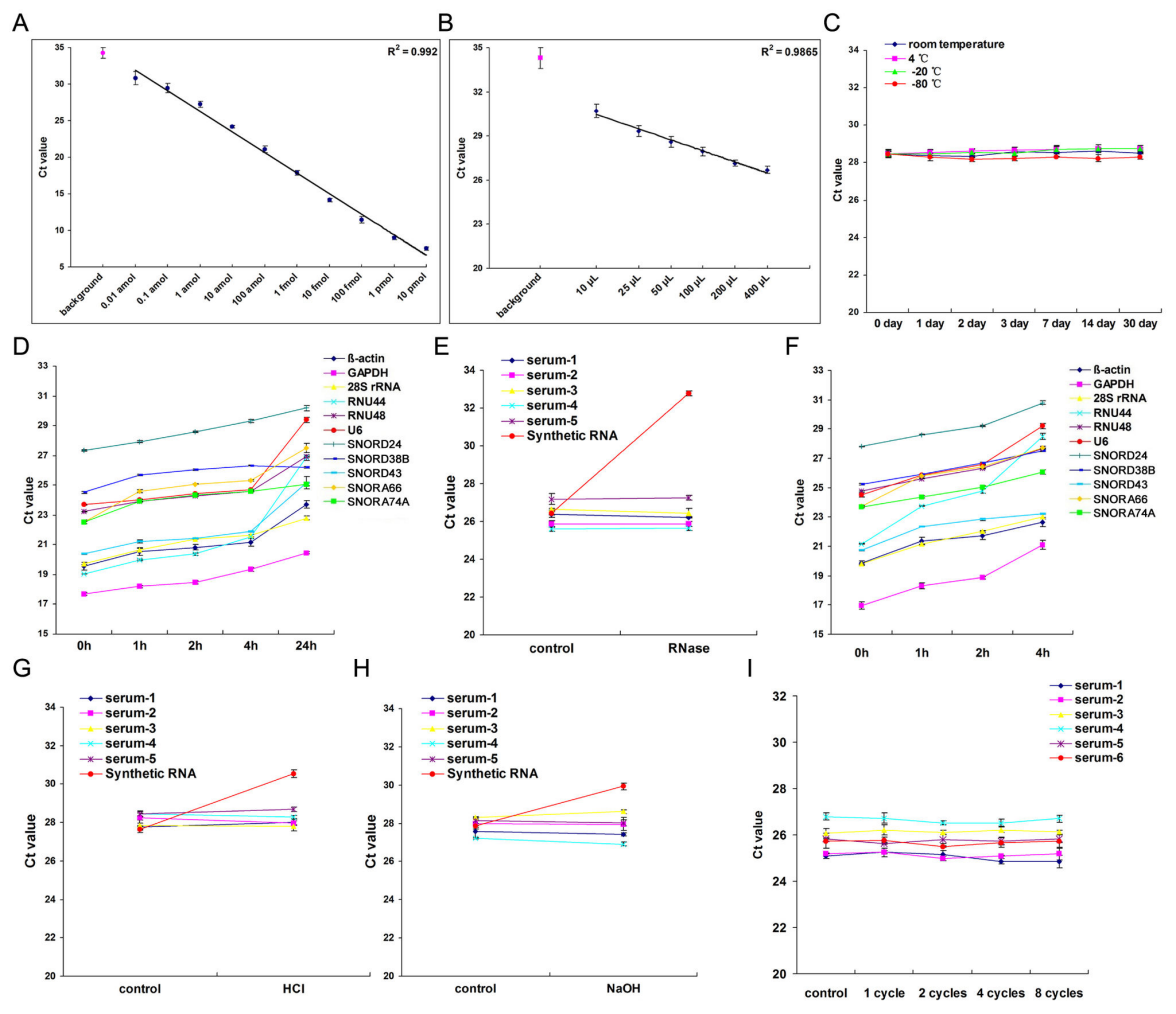
Characterization of the absolute concentration and the stability of let-7d/g/i in serum. (**A**) Dynamic range and sensitivity of the RT-qPCR assay for measuring let-7d/g/i (n = 5). Synthetic single-stranded let-7d/g/i ranging from 0.01 attomole (0.0033 attomole each, equivalent to 6×10^3^ copies in total) to 10 pmol (3.3 pmol each) were serially diluted over ten orders of magnitude and were assessed by the RT-qPCR assay. The resulting C_q_ values were plotted versus the amount of input let-7d/g/i to generate a standard curve. An assay using water instead of RNA for reverse-transcription was included as a negative control. (**B**) Correlation of serum volume to the C_q_ values (n = 5). Total RNA was extracted from different volumes of serum ranging from 10 µL to 400 µL. The levels of serum let-7d/g/i were assessed by RT-qPCR. The resulting C_q_ values were plotted versus the serum volume used for RNA extraction. An assay using water instead of RNA for reverse-transcription was included as a negative control. (**C**) Stability of let-7d/g/i in serum after extended storage (n = 5). Serum samples were equally divided and stored at room temperature, 4°C, -20°C or -80°C for 1, 2, 3, 7, 14 or 30 days. For each time point, total RNA was isolated and let-7d/g/i was measured by RT-qPCR assay. Storage at room temperature for 30 days yielded no apparent increase in C_q_ values. (**D**) Instability of other RNAs in serum (n = 5). Serum samples were equally divided and stored at room temperature for 1 to 24 h. For each time point, total RNA was isolated, and the levels of some large molecular weight RNA (β-actin, GAPDH and 28S rRNA) and snRNA/snoRNA (U6, RNU44, RNU48, SNORD24, SNORD38B, SNORD43, SNORA66 and SNORA74A) were measured by RT-qPCR. Storage at room temperature for 24 h resulted in an apparent increase of C_q_ values for these RNAs. (**E**) Stability of let-7d/g/i in serum after RNase digestion (n = 5). Serum samples were treated with 10 U/ml RNase A and 400 U/ml RNase T1 for 4 h at 37°C. After the treatment, the RNA was extracted from the serum, and the levels of let-7d/g/i were assessed by RT-qPCR. (**F**) Instability of other RNAs in serum after RNase digestion (n = 5). Serum samples were treated with 10 U/ml RNase A and 400 U/ml RNase T1 for 1, 2 or 4 h at 37°C. After the treatment, the RNA was extracted and the levels of the indicated RNAs were assessed by RT-qPCR assay. (**G** and **H**) Stability of let-7d/g/i under acidic or alkaline conditions (n = 5). Serum samples were incubated for 1 h under acidic (pH 2) or alkaline (pH 12) conditions. The levels of let-7d/g/i were assessed by RT-qPCR. (**I**) Stability of let-7d/g/i in serum following re-freezing and re-thawing of the samples (n = 6). Serum samples were subjected to eight freeze-thaw cycles.

We next evaluated the linear dynamic range and sensitivity of the RT-qPCR assay for measuring let-7d/g/i. Synthetic single-stranded let-7d/g/i was serially diluted and assessed by the RT-qPCR assay. Decreasing the amount of let-7d/g/i led to a corresponding increase in the mean C_q_ values, with a Pearson correlation coefficient (R) of 0.992 (Figure 4A). The results demonstrated that the let-7d/g/i RT-qPCR assay has a dynamic range of at least ten orders of magnitude and is capable of detecting as few as 0.01 attomole of let-7d/g/i (equivalent to 6,000 copies). Additionally, the expression levels of let-7d/g/i in RNA samples isolated from various volumes of serum were characterized using the RT-qPCR assay. The RT-qPCR assay showed excellent linearity between the serum volume and C_q_ value (R = 0.9865) (Figure 4B). These results demonstrate that let-7d/g/i in as little as 10 µL of serum can be efficiently detected and reliably compared across multiple samples. By referring to the standard curve, we calculated that the absolute concentration of let-7d/g/i in serum was 271.35 ± 21.48 fmol/L.

### The stability of let-7d/g/i in serum

To be used as a suitable reference gene in clinical tests, let-7d/g/i must be stable in serum for reasonable periods of time and preferably resistant to some harsh conditions, thereby allowing for routine processing of clinical samples. We first studied the stability of let-7d/g/i in sera stored for extended periods of time at different temperatures. No significant differences in C_q_ values were observed between the different storage time points (long-term versus short-term) or storage conditions (sub-zero versus high temperature) (Figure 4C). In contrast, large molecular weight RNA and snRNA/snoRNA were quickly degraded within 24 hours of storage at room temperature (Figure 4D). Furthermore, when serum samples were treated with RNase serum let-7d/g/i had considerable resistance to enzymatic cleavage (Figure 4E), but synthetic let-7d/g/i and other RNAs rapidly degraded (Figure 4F). The results suggest that RNase in serum can rapidly degrade large molecular weight RNA and snRNA/snoRNA but has much less of an effect on serum let-7d/g/i. Moreover, we found that the levels of let-7d/g/i in serum did not change substantially in acidic or alkaline conditions (Figure 4, G and H) and were barely affected by eight cycles of re-freezing and re-thawing of serum samples (Figure 4I). 

### Quantification of serum miRNAs is significantly influenced by different normalization approaches

**Figure 5:**
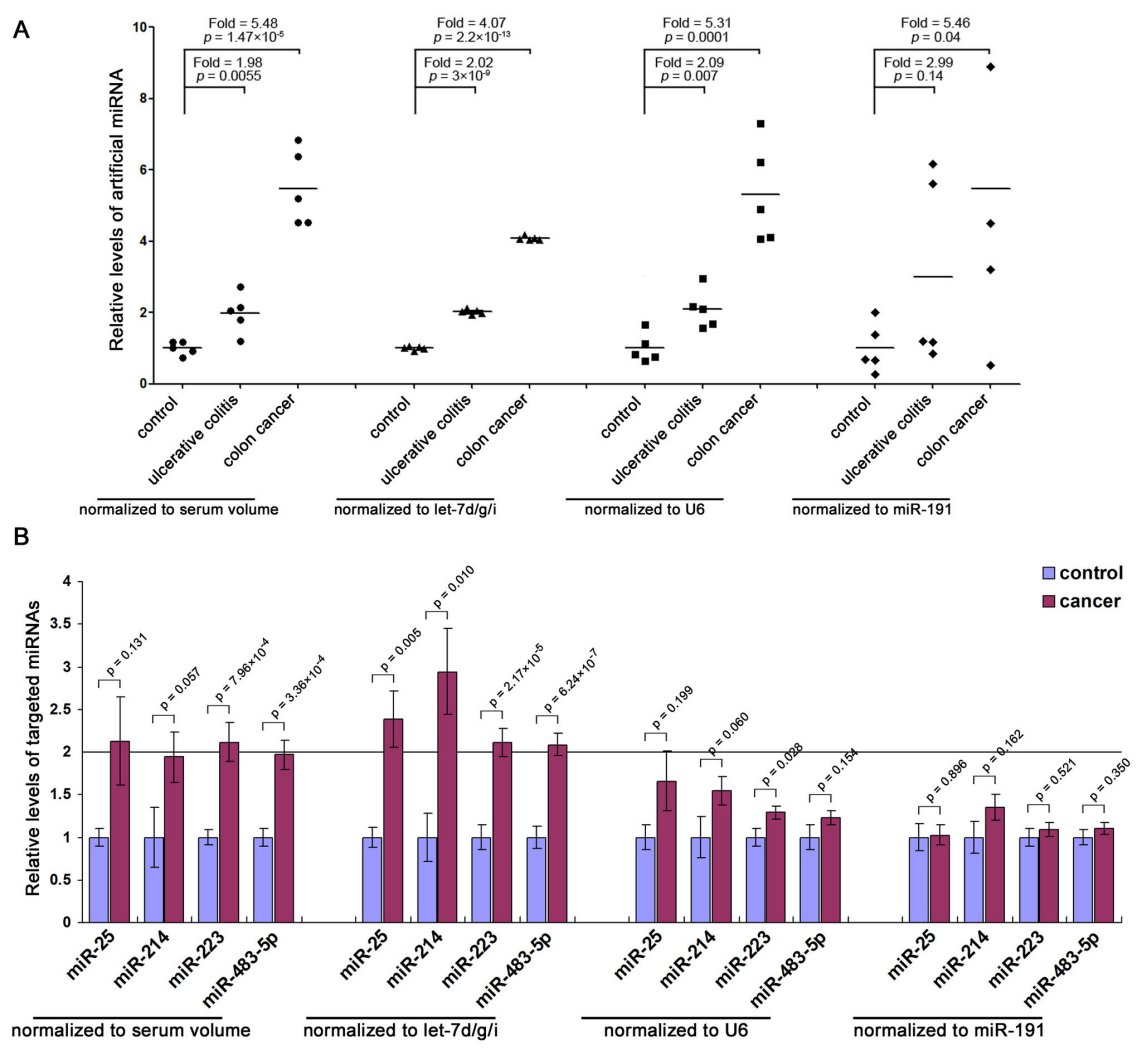
Effect of different normalization approaches on the levels of serum miRNAs. (**A**) Serum samples from healthy controls, ulcerative colitis patients and colon cancer patients were divided into three groups (n =5 in each group). After the initial denaturation steps, 1, 2 and 4 attomole of synthetic artificial miRNA (5’-GUGGAUUCCGUCUCGUUAG-3’) were spiked into 100 μL of serum of each group (1 attomole for control group, 2 attomole for ulcerative colitis group and 4 attomole for colon cancer group). After isolation of total RNA, the levels of artificial miRNA were assessed by RT-qPCR assay and were normalized to serum volume, let-7d/g/i, U6 or miR-191, respectively. Relative levels were calculated using the 2^-△△Cq^ method and were shown by dot plots. Significance was calculated by t-test. (**B**) Expression levels of miR-25, miR-214, miR-223 and miR-483-5p were measured in serum from cancer patients (n = 84) and healthy controls (n = 41) by RT-qPCR and were normalized to serum volume, let-7d/g/i, U6 or miR-191. Relative levels were calculated using the 2^-△△Cq^ method and are presented as mean fold changes ± standard errors. Significance was calculated by t-test.

To demonstrate that our proposed method is the best approach to control for variations in RNA recovery and amplification efficiency, we test the accuracy of let-7d/g/i as a reference gene by using exogeneous miRNA (spiked-in miRNA) as target. A 22-nt artificial miRNA which showed no sequence homology with endogenous miRNAs was spiked into three groups of serum samples (in the ratio of 1:2:4) after the addition of denaturing solution. Then, total RNA was isolated and the relative levels of artificial miRNA were calculated by normalization with serum volume, let-7d/g/i, U6 or miR-191, individually. Although all normalization approaches showed gradually elevation of artificial miRNA in groups with more added artificial miRNA, normalization with let-7d/g/i produced the most consistent results compared with the initial input (Figure 5A). The results suggest that normalization to let-7d/g/i is a superior normalization method as it corrects experimental variations better than existing methods and achieves accurate identification where others do not. Furthermore, we selected endogenous miR-25, miR-214, miR-223 and miR-483-5p as targets because they are well-characterized oncogenic miRNAs that have been shown to be elevated in sera from cancer patients [5,35,37]. We assessed the relative levels of these miRNAs in the sera of cancer patients and healthy controls by normalizing to serum volume, let-7d/g/i, U6 or miR-191, respectively. Only those miRNAs with a mean fold change > 2 and p-value < 0.05 were considered to be significantly upregulated. Normalization with let-7d/g/i revealed significant upregulation of miR-25, miR-214, miR-223 and miR-483-5p in serum from cancer patients compared with normal controls (Figure 5B). The direction of the fold change after normalization to serum volume was in agreement with the normalization with let-7d/g/i, but only miR-223 was shown to be significantly higher in serum from cancer patients (Figure 5B). However, normalization with U6 or miR-191 revealed no significant differences in miR-25, miR-214, miR-223 or miR-483-5p levels in serum from cancer patients versus controls (Figure 5B). The results further demonstrate that different normalization strategies significantly influence the consequence: the use of stable genes for normalization will improve sensitivity and reproducibility, whereas the choice of unstable reference genes can lead to inaccurate results.

## Discussion

Ideal endogenous reference genes should do the following: (1) exhibit stable expression across all samples and experimental conditions; (2) have comparable abundance to the targets of interest; and (3) share similar properties as the targets, such as stability, size and purification procedure [38]. To date, the selection of reference genes to normalize circulating miRNAs is still rather empirical, and no reference genes that fulfill the above-mentioned criteria are currently available. Because the yield of total RNA from small-volume serum samples (i.e., 100 µL) was below the limit of accurate quantification by spectrophotometry, the inclusion of an endogenous reference gene is indispensable for adjusting for technical variations in the RNA recovery procedure. Bias in sample collection, storage and processing also affects the accuracy and reliability of the quantitative analysis of circulating miRNAs. As such, systematic selection and validation of suitable reference genes for quantification of circulating miRNAs is needed, especially in diseases such as cancer where the global expression of circulating miRNAs may be dramatically altered in the context of rapid physiological and pathological changes.

Although several genes are traditionally utilized as endogenous reference genes for tissue/cell miRNAs, it is not reasonable to use these genes to normalize circulating miRNA levels because they are not miRNAs and may not be representative of the miRNA fraction. In addition, the efficiency of their extraction, reverse transcription and PCR amplification may differ from that of circulating miRNAs. Therefore, these genes may not be an ideal choice. Indeed, our study clearly demonstrates that these genes varied greatly in serum and were unstable even after short-term storage (Figure 4).

In previous studies, synthetic non-human (e.g., *C. elegans*) miRNAs have been used as spike-in controls to normalize the levels of circulating miRNAs [6]. These molecules can provide a reference for normalization of the technical variability in RNA extraction. However, spike-in controls do not correct for variability in sample collection and therefore cannot improve assay precision. Indeed, it has been reported that the spiked exogenous *C. elegans* miRNAs, including cel-miR-39, cel-miR-54 and cel-miR-238, did not significantly improve assay precision [39]. Therefore, spike-in controls are also not an ideal choice.

The high stability of circulating miRNAs in human body fluids raises important and intriguing questions regarding the mechanism by which miRNAs are protected from digestion. Two models have been proposed to account for this: (1) circulating miRNAs are protected by packaging inside microvesicles [40], and (2) circulating miRNAs are protected via association with proteins such as Argonaute2 (Ago2), high-density lipoprotein and nucleophosmin 1 (NPM1) [41]. From this point of view, circulating miRNAs represent a unique class of molecules that differ from other types of RNAs in serum. Therefore, circulating miRNAs themselves may be the sole suitable reference genes for normalization of circulating miRNAs, as they share similar properties, such as stability and purification procedure. However, the reference circulating miRNAs must be carefully selected and systematically validated to avoid inaccurate normalization. For example, studies have been performed using circulating miR-16 as a reference gene under the assumption that its levels are stable across experimental conditions [4,31-33]. However, a systematic evaluation of miR-16 as a reference gene for normalization has not been published. In fact, it has been reported that endogenous miR-16 was a poor normalizing factor [39]. By employing SBS technology and RT-qPCR assay, we selected and validated the combination of let-7d, let-7g and let-7i as the best reference gene for normalization of serum miRNAs (Figure 2 and 3). Furthermore, we systematically demonstrate that normalization to let-7d/g/i is a superior normalization method as it corrects experimental variations better than existing methods and achieves accurate identification where others do not (Figure 5). However, the reference genes identified currently were different from those found in previous studies [14,42], although these studies also used similar methods and algorithms (e.g., geNorm and NormFinder) to select suitable reference genes. We think that this inconsistency may be due to the differences in study design, disease type, sample size and methodology. For example, the aim of this study is to identify the common reference gene for normalization of serum miRNAs; thus, we investigate serum miRNAs in a large sample set including more than 2000 serum samples across healthy controls and patients with a variety of different diseases. In contrast, the previous studies focused on a specific disease and therefore investigated miRNAs only in a small patient cohort. Furthermore, we screened the levels of all miRNAs in serum by SBS technology, while the previous studies only assessed limited miRNAs. Nevertheless, all of these studies demonstrated that reference gene choice for circulating miRNA analysis has a great effect on the study outcome, and that it is necessary to choose a suitable reference for reliable normalization of circulating miRNAs.

In summary, our findings constitute the first report describing the rigorous identification and validation of suitable reference genes for normalization of miRNAs in serum. This has important implications for proper experimental design and accurate data interpretation.

## Supporting Information

Figure S1 **Discrimination ability of the RT-qPCR assay for individual members of the let-7 family.** Because the let-7 family members differ by only a single or a few nucleotides, it should be ensured that the RT-qPCR assays can discriminate each members of the let-7 family without interference. To this end, 10 attomole of synthetic single-stranded let-7a, let-7b, let-7c, let-7d, let-7e, let-7f, let-7g and let-7i were individually assessed by the RT-qPCR assay; each assay was examined against a targeted let-7 member and the remaining let-7 species. Relative detection rate was calculated based on the C_q_ values between perfectly matched and mismatched targets, assuming 100% efficiency for the perfect match. The results showed that RT-qPCR assays targeting the matched let-7 species produced C_q_ values much lower than those for mismatched; mismatched targets would contribute < 1% background signal to the assay of the targeted let-7 species.
(DOC)

Figure S2 **Standard curves for let-7d, let-7g and let-7i.** The standard curves were generated by RT-qPCR amplifying with 10^4^, 10^3^, 10^2^, 10, 1 or 0.1 attomole of synthetic single-stranded let-7d, let-7g or let-7i, respectively (n = 5). The resulting C_q_ values were plotted against the logarithm of the input amount of let-7d, let-7g and let-7i. The slope and intercept for let-7d, let-7g or let-7i were about −3.4 and 27.8, indicating approximately equal amplification efficiency.
(DOC)

Methods S1 **Detailed methodology for RNA isolation, SBS technology, RT-qPCR and data analysis.**
(DOC)

Table S1 **Primer sequences of the housekeeping genes.**
(DOC)
