# Supplementary figures and images for: A Combination of Let-7d, Let-7g and Let-7i Serves as a Stable Reference for Normalization of Serum microRNAs
Source: PLoS One. 2013 Nov 5;8(11):e79652. doi: 10.1371/journal.pone.0079652 (PMC3818225; doi:10.1371/journal.pone.0079652)

**Figure S1.** **Discrimination ability of the RT-qPCR assay for individual members of the let-7 family.**


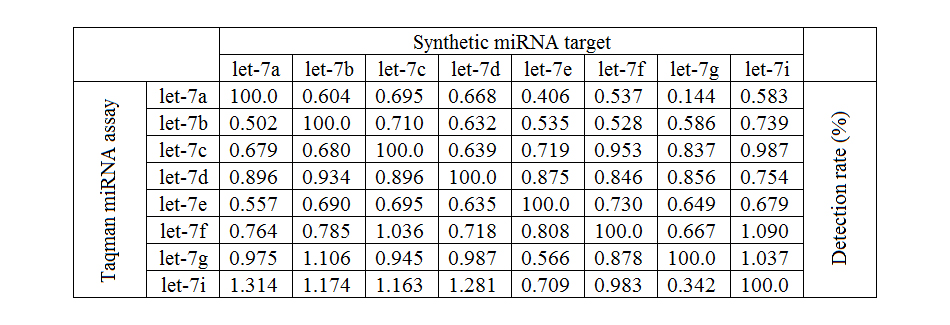

Supplement: Figure S1 — Discrimination ability of the RT-qPCR assay for individual members of the let-7 family. Because the let-7 family members differ by only a single or a few nucleotides, it should be ensured that the RT-qPCR assays can discriminate each members of the let-7 family without interference. To this end, 10 attomole of synthetic single-stranded let-7a, let-7b, let-7c, let-7d, let-7e, let-7f, let-7g and let-7i were individually assessed by the RT-qPCR assay; each assay was examined against a targeted let-7 member and the remaining let-7 species. Relative detection rate was calculated based on the Cq values between perfectly matched and mismatched targets, assuming 100% efficiency for the perfect match. The results showed that RT-qPCR assays targeting the matched let-7 species produced Cq values much lower than those for mismatched; mismatched targets would contribute < 1% background signal to the assay of the targeted let-7 species. (DOC) [file pone.0079652.s001.doc]

**Figure S2. Standard curves for let-7d, let-7g and let-7i.**


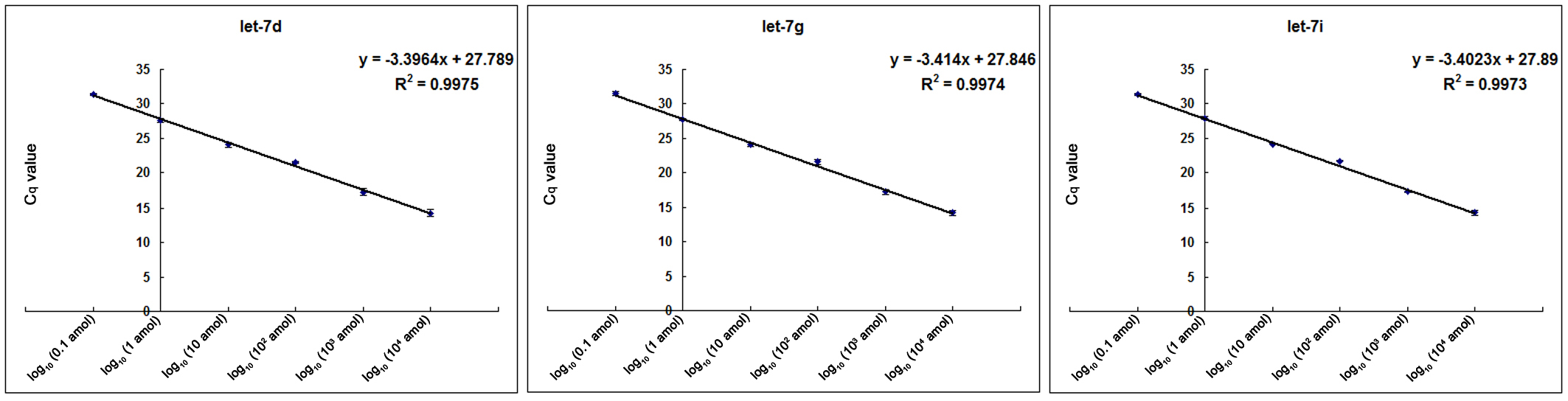

Supplement: Figure S2 — Standard curves for let-7d, let-7g and let-7i. The standard curves were generated by RT-qPCR amplifying with 104, 103, 102, 10, 1 or 0.1 attomole of synthetic single-stranded let-7d, let-7g or let-7i, respectively (n = 5). The resulting Cq values were plotted against the logarithm of the input amount of let-7d, let-7g and let-7i. The slope and intercept for let-7d, let-7g or let-7i were about −3.4 and 27.8, indicating approximately equal amplification efficiency. (DOC) [file pone.0079652.s002.doc]
