# Supplementary material for: A Combination of Let-7d, Let-7g and Let-7i Serves as a Stable Reference for Normalization of Serum microRNAs
Source: PLoS One. 2013 Nov 5;8(11):e79652. doi: 10.1371/journal.pone.0079652 (PMC3818225; doi:10.1371/journal.pone.0079652)
Supplement: Methods S1 — Detailed methodology for RNA isolation, SBS technology, RT-qPCR and data analysis. (DOC) [file pone.0079652.s003.doc]

**Method S1. Detailed methodology for RNA isolation, SBS technology, RT-qPCR and data analysis.**

**Profiling circulating miRNAs using sequencing by synthesis (SBS) technology**

Total RNA was extracted from 50 mL of pooled serum using the TRIzol Reagent (Invitrogen, Carlsbad, CA, USA) according to the manufacturer’s instructions. The total RNA were then subjected to 15% denaturing polyacrylamide gel electrophoresis, and the 18-30 nt size range of RNA was isolated from the gel and purified. After purification, small RNAs were ligated to a 5’ RNA adapter (5’-GUUCAGAGUUCUACAGUCCGACGAUC-3’), followed by another gel purification and ligated to a 3’ RNA adapter (5’-pUCGUAUGCCGUCUUCUGCUUGidT-3’, idT is an inverted deoxythymidine). The purified small RNAs were reverse transcribed using Illumina’s small RNA RT-Primer (5’-CAAGCAGAAGACGGCATACGA-3’) and amplified by a 17 cycle PCR using Illumina’s small RNA primer set (5’-CAAGCAGAAGACGGCATACGA-3’ and 5’-AATGATACGGCGACCACCGA-3’). PCR products were purified and quantified to produce libraries and were sequenced using an Illumina Genome Analyzer at Shenzhen Huada Gene Sci-Tech Company (Shenzhen, China). The image files generated by the sequencer were then processed to produce digital data. The subsequent procedures included summarizing data production, evaluating sequence quality and depth, calculating length distribution of small RNAs and filtering contaminated reads. After masking the adaptor sequences, the clean reads were aligned against the miRBase database 16.0 by the Smith-Waterman algorithm. Only a candidate with an identical sequence and length compared to the reference miRNA was counted as a miRNA match. Finally, the total sequencing frequency of each sample was adjusted to an equal scale of 1,000,000. All SBS runs performed at the Illumina Genome Analyzer include a control lane for measuring run quality. In the present study, all of the control lanes on the Illumina Genome Analyzer System meet acceptable quality standards, and then the results are deemed to be of acceptable quality.

**Quantification of circulating miRNAs by RT-qPCR assay**

Total RNA was extracted from 100 μL of serum with a one-step phenol/chloroform purification protocol. Briefly, 100 μL serum was sequentially mixed with 300 μL RNase-free water, 200 μL acid phenol and 200 μL chloroform. The mixture was vortex-mixed vigorously and incubated at room temperature for 15 min. After phase separation, the aqueous layer was mixed with 1.5 volumes of isopropyl alcohol and 0.1 volumes of 3 mol/L sodium acetate (pH 5.3). This solution was stored at -20°C for 1 h. The RNA pellet was collected by centrifugation at 16,000 g for 20 min at 4°C. The resulting RNA pellet was washed once with 75% ethanol and dried for 10 min at room temperature. Finally, the pellet was dissolved in 20 μL of RNase-free water and stored at -80°C until further analysis. RNA concentration and quality (OD260/OD280 ratio) were measured using a Qubit fluorometer (Invitrogen) and a Quant-iT RNA Assay Kit (Invitrogen) according to the manufacturer’s instructions. In general, the yield of total RNA extraction was 50-100 ng per 100 μl serum.

Quantification of circulating miRNAs was carried out using a Taqman miRNA PCR kit (Applied Biosystems, Foster City, CA, USA) according to the manufacturer’s instructions. Briefly, 5 μL of total RNA was reverse-transcribed to cDNA using AMV reverse transcriptase (TaKaRa, Dalian, China) and stem-loop RT primers (Applied Biosystems). Real-time PCR was performed using TaqMan miRNA probes (Applied Biosystems) on the Applied Biosystems 7300 Sequence Detection System (Applied Biosystems). All reactions, including the no-template controls, were run in triplicate. After the reactions, the C_q_ values were determined using the fixed threshold settings. Each miRNA was reverse transcribed and amplified separately, and only one miRNA could be tested using 100 μL serum. For quantification of more miRNAs, more serum samples were used.

**Quantification of housekeeping genes**

Eleven housekeeping genes were selected, including β-actin, GAPDH, 28S rRNA, U6, RNU44, RNU48, SNORD24, SNORD38B, SNORD43, SNORA66 and SNORA74A. Gene sequences were obtained from the NCBI GenBank database. Primers were designed using the primer analysis software Primer Express v3.0 (Applied Biosystems). For each gene, a minimum of three pairs of primers were designed. Primers with best amplification efficiency were selected and used in further studies (Supplementary Table 1).

Total RNA was extracted from 100 μL of serum as described above. Then 5 μL of total RNA was reverse transcribed to cDNA using AMV reverse transcriptase (TaKaRa) and reverse primer. Real-time RT-PCR was performed on an Applied Biosystems 7300 Sequence Detection System (Applied Biosystems) using the SYBR Green PCR Master Mix (Applied Biosystems). The Cq values were determined using the fixed threshold settings. To verify that the used primer pair produced only a single product, a dissociation protocol was added after thermocycling, determining dissociation of the PCR products from 65°C to 95°C. All reactions, including no-template controls, were performed in triplicate.

**Analysis of the expression stability of candidate reference genes**

Two programs, geNorm and NormFinder, were applied to analyze the expression stability of candidate reference genes. SBS reads were used directly for stability calculations, and C_q_ values were transformed to relative quantities using the 2^-△△Cq^ method. The geNorm software provides a ranking of the tested genes based on their expression stability (M value), determining the two most stable reference genes or a combination of multiple stable genes for normalization. The value M represents the mean pairwise variation of one candidate reference gene compared to all other reference genes in the test panel. Genes with the lowest M values are considered the most stable. The stability ranking of each candidate gene is determined by stepwise exclusion of the gene with highest M value, followed by recalculation of average expression stability for the remaining genes until the two most stable genes are found. In addition, the geNorm program can determine the optimal number of reference genes required for accurate normalization by calculating the pairwise variation (V_n_/V_n+1_) between two sequential normalization factors (NF_n_ and NF_n+1_). A large value of variation (> 0.15) means that adding another reference gene is necessary for a more reliable normalization. If the variation V_n_/V_n+1_ drops below the recommended threshold of 0.15, inclusion of additional reference genes is considered to be unnecessary. NormFinder is a model-based approach that determines the expression stability of candidate reference genes according to their group origin (e.g., tumor versus normal). This approach determines the inter- and intra-group variation and combines both results in a stability value for each gene. According to this algorithm, genes with the lowest stability will be ranked highest. In the current study, two groups corresponding to different types of samples (disease versus control) were analyzed by NormFinder.

**Literature screening**

We conducted a PubMed search of the published literature to identify traditionally utilized reference genes in miRNA research. As this is a very broad topic, we narrowed the search strategy by limiting our consideration of articles by publication date (from 2006 to 2011) and language (English). Keywords included: “microRNA/miRNA” and “reference gene/housekeeping gene/internal control/normalizer”. Finally, 5 genes (GAPDH, β-actin, U6, RNU44 and RNU48) and 4 miRNAs (miR-16, miR-191, miR-103 and miR-23a) were selected, based on the frequency recording during literature screening.
