# Supplementary material for: A Combination of Let-7d, Let-7g and Let-7i Serves as a Stable Reference for Normalization of Serum microRNAs
Source: PLoS One. 2013 Nov 5;8(11):e79652. doi: 10.1371/journal.pone.0079652 (PMC3818225; doi:10.1371/journal.pone.0079652)
Supplement: Table S1 — Primer sequences of the housekeeping genes. (DOC) [file pone.0079652.s004.doc]

**Table S1. Primer sequences of the housekeeping genes.**

| Gene name | Official symbol | Accession number | Organism | Forward primer (5’-3’) | Reverse primer (5’-3’) | Product (bp) |
| --- | --- | --- | --- | --- | --- | --- |
| β-actin | ACTB | NM_001101 | Human | CTCCATCCTGGCCTCGCTGT | GCTGTCACCTTCACCGTTCC | 268 |
| GAPDH | GAPDH | NM_001256799 | Human | AGAAGGCTGGGGCTCATTTG | AGGGGCCATCCACAGTCTTC | 258 |
| 28S rRNA | RNA28S5 | NR_003287 | Human | GGCTACCCACCCGACCCGTCTTGAAACA | ACGTCAGGACCGCTACGGACCTCCACCA | 298 |
| U6 | RNU6-1 | NR_004394 | Human | CTCGCTTCGGCAGCACA | AACGCTTCACGAATTTGCGT | 94 |
| RNU44 | SNORD44 | NR_002750 | Human | GATGATGATAAGCAAATGCTGACTGAAC | GTTAGAGCTAATTAAGACCTTCATGTTC | 52 |
| RNU48 | SNORD48 | NR_002745 | Human | TGATGATGACCCCAGGTAACTCTGAGTG | GTCAGAGCGCTGCGGTGATGGCATCAGC | 60 |
|  | SNORD24 | NR_002447 | Human | TGATGTAAAAGAATATTTGCTATCTGAG | ATCAGCGATCTTGGTGGTTTAAAATGTC | 66 |
|  | SNORD38B | NR_001457 | Human | TCAGTGATGAAAACTTTGTCCAGTTCTG | TTTATCTTCACTTACTGTCAGTAGCAGA | 52 |
|  | SNORD43 | NR_002439 | Human | CAGATGATGAACTTATTGACGGG | ATCAGAACGTGACAATCAGCAC | 59 |
|  | SNORA66 | NR_002444 | Human | CAAAAGTAACTGTGGTGATGGAAATGTG | AGGATAGAAAGAACCACCTCAGTAGTGT | 66 |
|  | SNORA74A | NR_002915 | Human | CTTTATTGAGGTTTGGCTCCAAGCACTG | CACCCAGACTAGGATCAACTCCACAGGA | 116 |
